# Acute stent placement for dynamic airway collapse after cardiac surgery in congenital heart disease with bronchial stenosis: A case report

**DOI:** 10.1097/MD.0000000000044213

**Published:** 2025-09-05

**Authors:** Huanyu Qin, Nan Jiang, Siyang Qin, Yipeng Tang, Tongyun Chen

**Affiliations:** a Department of Cardiac Surgery, Chest Hospital, Tianjin University, Tianjin, China.

**Keywords:** airway stent, cardiac surgery, case report, tracheostenosis

## Abstract

**Rationale:**

Tracheomalacia, typically seen in relapsing polychondritis,^[1]^ is rarely reported in association with congenital heart disease (CHD). In patients with pulmonary hypoperfusion-type CHD, surgical repair results in a rapid increase in pulmonary blood flow, predisposing them to mucus retention, airway obstruction, and respiratory distress. We describe acute airway collapse in a patient with double outlet right ventricle and congenital bronchial stenosis following cardiac repair. Surgically augmented pulmonary flow critically narrowed the stenotic airway, rapidly reversed by bronchoscopic stenting. We introduce the concept of “hemodynamic-aggravated airway compromise” and advocate for protocolized monitoring in CHD-airway comorbidity cases.

**Patient concerns:**

A 19-year-old male with double outlet right ventricle and pulmonary stenosis underwent a central aortopulmonary shunt. Asymptomatic left main bronchial stenosis was incidentally found on preoperative CT. Four hours post-extubation, he developed acute respiratory distress. A bronchial stent was placed on postoperative day 10, resulting in marked clinical improvement and eventual discharge.

**Diagnoses:**

Stenosis of the left main bronchus and left upper lobe bronchus was noted on preoperative chest CT. Postoperative increases in pulmonary blood flow and mucus retention were thought to contribute to secondary airway obstruction. Bronchoscopy demonstrated significant luminal obstruction of the bronchus.

**Interventions:**

An airway stent was successfully deployed in the left main bronchus under bronchoscopic guidance. The patient recovered with comprehensive management including airway care, antimicrobial therapy, and inotropic support.

**Outcomes:**

Spontaneous breathing resumed on postoperative day 4 following stent placement; decannulation occurred day 10, and multidisciplinary discharge occurred on day 18 with stable cardiopulmonary status.

**Lessons:**

In patients who experience postoperative failure to liberate from mechanical ventilation accompanied by recurrent mucus retention, the possibility of underlying structural airway abnormalities should be carefully considered. Early evaluation with bronchoscopy and cross-sectional airway imaging is warranted for timely and accurate diagnosis. Once the anatomic obstruction is confirmed, bronchial stent placement represents a feasible, effective, and controllable therapeutic strategy. Rigorous pre-procedural assessment of indications and vigilant postoperative surveillance are essential to minimize the risk of stent-related complications.

## 1. Introduction

Congenital heart disease (CHD) associated with congenital airway stenosis is relatively rare, and the mechanisms underlying postoperative airway decompensation remain unclear. Existing literature has primarily focused on complications such as pulmonary hypertension^[2,3]^ or anastomotic stenosis after CHD surgery. In contrast, studies addressing the role of abrupt pulmonary blood flow changes in precipitating airway decompensation are limited. Traditional management strategies often lead to delays in timely intervention.

In patients with pulmonary hypoperfusion-type CHD, surgical correction typically results in a significant increase in pulmonary blood flow, predisposing them to mucus retention, airway obstruction, and respiratory distress. We report a case of double outlet right ventricle (DORV) with congenital bronchial stenosis. The patient had no obvious respiratory symptoms preoperatively but developed acute respiratory failure after surgery. Bronchoscopic airway stent placement led to rapid symptom relief and successful recovery.

Unlike previous reports, this case highlights the dynamic impact of postoperative pulmonary hemodynamic changes on airway patency and emphasizes the importance of optimal timing for stent intervention. It also underscores the effectiveness of minimally invasive therapy in the acute phase. A review of the relevant literature is included to explore the clinical features and management strategies of airway stenosis following congenital cardiac surgery.

## 2. Case report

A 19-year-old male was admitted due to a 19-year history of cyanosis involving the lips and extremities. He was diagnosed with CHD at birth in a local hospital but received no prior treatment. He had hypoxic spells during childhood and poor exercise tolerance.

On admission, physical examination revealed marked central and peripheral cyanosis, and positive clubbing of the fingers and toes. His vital signs were: pulse rate 105 bpm, blood pressure 123/72 mm Hg (left upper limb), and oxygen saturation (SpO_2_) was 83% (left arm), 90% (left foot), 82% (right arm), and 84% (right foot). Heart rate was 105 bpm with a regular rhythm. A grade II/VI early systolic rough murmur was auscultated at the left sternal border between the 2nd and 4th intercostal spaces, with limited radiation and diminished pulmonary component of the second heart sound. There was no edema in the lower limbs.

**Figure 1.**
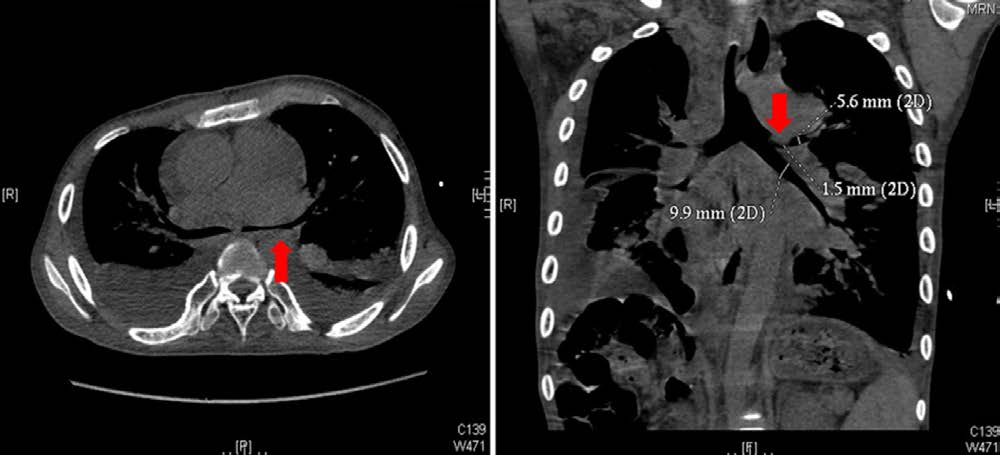
Axial mediastinal window CT (A) and coronal reconstructed CT (B) demonstrate narrowing of the anteroposterior diameter of the left main bronchus and the orifice of the left upper lobe bronchus (red arrows). CT = computed tomography.

Cardiac CT revealed situs solitus with left-sided heart, right atrial isomerism (asplenia syndrome), DORV, large atrial and ventricular septal defects, and both valvular and subvalvular pulmonary stenosis. The great arteries were malposed with the aorta anterior and rightward to the pulmonary artery. A large atrial septal defect measuring 2.2 cm and a large ventricular septal defect measuring 3.3 cm were present. Subvalvular pulmonary stenosis was noted, with the narrowest outflow tract diameter at 7 mm. The main pulmonary artery measured 2.3 cm, left pulmonary artery 1.1 cm, and right pulmonary artery was hypoplastic at 0.7 cm. The McGoon index was calculated as 1.1, indicating insufficient pulmonary artery development. Pulmonary venous drainage was markedly abnormal. The left pulmonary veins drained into a persistent left superior vena cava that emptied into the left atrium, while the right pulmonary veins converged into a common trunk draining into the hepatic veins. This configuration was consistent with partial anomalous pulmonary venous connection and systemic venous anomalies.Preoperative chest CT also showed narrowing of the left main bronchus (anteroposterior diameter 2.1 mm) and stenosis at the orifice of the left upper lobe bronchus (approximately 1.5 mm) (Fig. 1). Notably,The patient had no respiratory symptoms before surgery.

Transthoracic echocardiography demonstrated showed a dominant right ventricle with marked wall thickening, whereas the left ventricle was hypoplastic with an end-diastolic volume of approximately 64.4 mL. Although left ventricular function was preserved, the chamber size was insufficient to support a biventricular circulation.

Given the unbalanced ventricles, severe pulmonary outflow tract obstruction, right pulmonary artery hypoplasia, and complex anomalous pulmonary venous return, the patient was not considered a candidate for complete biventricular repair or Fontan-type univentricular palliation. A central aortopulmonary shunt using a 6 mm pericardial graft was therefore chosen as a palliative approach to improve oxygenation under general anesthesia. The procedure was uneventful, and he was transferred to the intensive care unit for postoperative management, including mechanical ventilation, inotropic support, diuretics, antibiotics, and general supportive care.

On postoperative day 1, the patient resumed spontaneous breathing and was extubated. However, 4 hours after extubation, he developed difficulty expectorating sputum and experienced respiratory distress. Arterial blood gas analysis showed a PaO_2_of 49 mm Hg and a PaCO_2_ of 100 mm Hg. Emergency re-intubation was performed, and mechanical ventilation was restarted. Due to repeated failure to wean from ventilatory support, a tracheostomy was performed on postoperative day 8. Given the preoperative chest CT findings of congenital stenosis in the left main bronchus and left upper lobe bronchus, along with postoperative factors including increased pulmonary blood flow and mucus retention, secondary airway obstruction was considered. On postoperative day 10, the patient underwent bronchoscopic placement of a stent in the left main bronchus.

**Figure 2.**
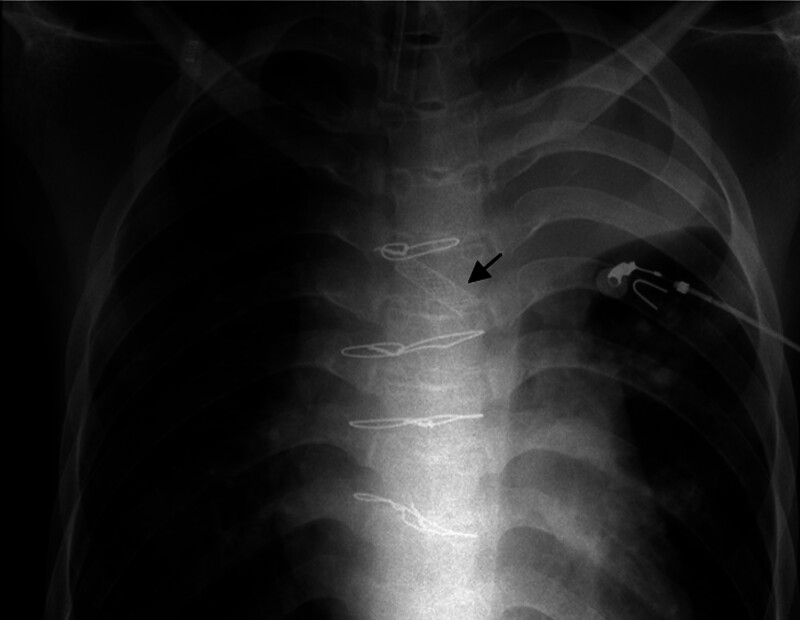
Post-stent chest X-ray showing the implanted stent (black arrow).

During the procedure, the patient was placed in a supine position with a neck extension. Topical pharyngeal anesthesia and nebulized inhalation anesthesia were administered. A guidewire was inserted through the working channel of the bronchoscope, advanced past the stenotic segment into the distal left main bronchus, and left in place while the bronchoscope was withdrawn. A delivery system preloaded with a nitinol stent was then introduced through the tracheostomy site and advanced under direct visualization to the proximal end of the stenosis. After confirming the correct positioning, the stent was deployed slowly. The stent expanded well, and the airway was successfully reopened (Fig. 2). Postoperatively, the patient continued to receive comprehensive treatment, including inotropes, antibiotics, and intensive airway management. His respiratory status improved markedly, with good re-expansion of the left lung and effective spontaneous sputum clearance. No complications such as bronchial hemorrhage or aspiration were observed. By postoperative day 4 after stent placement, the patient began to regain spontaneous breathing, and by day 10, he was successfully weaned from the ventilator, and the tracheostomy tube was closed. He was discharged on postoperative day 18 with satisfactory clinical and radiographic recovery.

## 3. Discussion and conclusions

CHD complicated by congenital airway stenosis is rare but clinically significant. In such patients, abrupt postoperative changes in pulmonary hemodynamics can precipitate acute airway decompensation, potentially resulting in life-threatening respiratory obstruction.^[4]^ In our case, the patient had no apparent respiratory symptoms before surgery, despite preoperative imaging identifying congenital narrowing of the left main bronchus. However, shortly after extubation, he developed ineffective sputum clearance and progressive respiratory distress. Initially, these symptoms were attributed to postoperative secretion retention and cardiac-related factors. Yet, conventional airway management strategies, including bronchoscopy-guided suctioning and nebulized therapy, failed to resolve the obstruction. Subsequent imaging and bronchoscopy revealed that the primary etiology was structural stenosis of the left main bronchus, with retained secretions as a secondary aggravating factor. The patient was diagnosed with DORV (Fallot-type) accompanied by valvular and subvalvular pulmonary stenosis. The patient also had poor pulmonary artery development, with a McGoon index of 1.1, indicating long-term preoperative pulmonary hypoperfusion. After undergoing central aortopulmonary shunt surgery using a 6 mm pericardial tube graft anastomosed between the aorta and main pulmonary artery, pulmonary perfusion increased abruptly, while left heart function remained relatively insufficient. This resulted in pulmonary congestion and increased secretions. Additionally, congenital stenosis of the left main bronchus and the patient’s poor early postoperative physical condition impaired effective sputum clearance, leading to worsening airway obstruction. Conventional treatments such as bronchoscopic suctioning and nebulization were ineffective, and the patient remained ventilator-dependent. Bronchoscopy confirmed stenosis of the left main bronchus, and bronchial stent implantation was subsequently performed. Airway stenting has been proven to be a safe and effective intervention^[5]^ for advanced airway collapse or stenosis, with advantages including technical simplicity and accurate positioning.^[6]^

Currently, 2 main types of stents are widely used for airway interventions^[7]^: covered stents and uncovered metallic mesh stents.^[8,9]^ In this case, an uncovered metallic mesh stent was utilized. This type of stent allows exposure of the bronchial cilia, thereby preserving mucociliary clearance, and is also characterized by high mechanical strength and corrosion resistance. However, some studies have suggested that uncovered stents may irritate the tracheal mucosa, leading to granulation tissue formation and fibrosis, which can result in restenosis. The long-term efficacy of tracheal dilation using such stents remains to be further evaluated. Various methods are available for bronchial stent placement, including tracheostomy, fluoroscopic guidance, and bronchoscopic guidance.^[10]^ Among these, bronchoscopic stent placement is currently considered the safest and most reliable technique 11. In the present case, the stent was placed via tracheostomy under bronchoscopic guidance. This approach enables real-time visualization and adjustment of the stent position, and in the event of bleeding, allows for prompt suctioning and topical hemostatic intervention to prevent airway obstruction.^[11]^

The novelty of this study lies in its identification of a causal relationship between early postoperative failure to wean from mechanical ventilation and congenital bronchial stenosis in a patient with complex CHD. It highlights the importance of preoperative airway structural evaluation, particularly in children with pulmonary hypoplasia. In this case, the placement of a bronchial stent during the acute phase – under bronchoscopic guidance via a tracheostomy route – provided a feasible, safe, and effective intervention strategy for managing such conditions. This case underscores the need for heightened clinical vigilance regarding airway structural abnormalities in postoperative patients with persistent ventilator dependence or recurrent sputum retention. Early bronchoscopic and imaging assessments of the airway should be performed to establish a definitive diagnosis. Once identified, bronchial stenting offers a controllable and effective treatment option. However, strict preoperative screening for indications and close postoperative follow-up are essential to prevent stent-related complications.

## Author contributions

**Conceptualization:** Yipeng Tang.

**Project administration:** Nan Jiang.

**Resources:** Siyang Qin.

**Supervision:** Tongyun Chen.

**Writing – original draft:** Huanyu Qin.
